# Combining Statistical Analysis and Machine Learning for EEG Scalp Topograms Classification

**DOI:** 10.3389/fnsys.2021.716897

**Published:** 2021-11-16

**Authors:** Alexander Kuc, Sergey Korchagin, Vladimir A. Maksimenko, Natalia Shusharina, Alexander E. Hramov

**Affiliations:** ^1^Center for Neurotechnology and Machine Learning, Immanuel Kant Baltic Federal University, Kaliningrad, Russia; ^2^Department of Data Analysis and Machine Learning, Financial University Under the Government of the Russian Federation, Moscow, Russia; ^3^Institute of Information Technologies, Mathematics and Mechanics, Lobachevsky State University of Nizhny Novgorod, Nizhny Novgorod, Russia; ^4^Neuroscience and Cognitive Technology Laboratory, Innopolis University, Innopolis, Russia

**Keywords:** EEG topograms, convolutional neural network, CNN, ambiguous stimuli, pre-trained decoder

## Abstract

Incorporating brain-computer interfaces (BCIs) into daily life requires reducing the reliance of decoding algorithms on the calibration or enabling calibration with the minimal burden on the user. A potential solution could be a pre-trained decoder demonstrating a reasonable accuracy on the naive operators. Addressing this issue, we considered ambiguous stimuli classification tasks and trained an artificial neural network to classify brain responses to the stimuli of low and high ambiguity. We built a pre-trained classifier utilizing time-frequency features corresponding to the fundamental neurophysiological processes shared between subjects. To extract these features, we statistically contrasted electroencephalographic (EEG) spectral power between the classes in the representative group of subjects. As a result, the pre-trained classifier achieved 74% accuracy on the data of newly recruited subjects. Analysis of the literature suggested that a pre-trained classifier could help naive users to start using BCI bypassing training and further increased accuracy during the feedback session. Thus, our results contribute to using BCI during paralysis or limb amputation when there is no explicit user-generated kinematic output to properly train a decoder. In machine learning, our approach may facilitate the development of transfer learning (TL) methods for addressing the cross-subject problem. It allows extracting the interpretable feature subspace from the source data (the representative group of subjects) related to the target data (a naive user), preventing the negative transfer in the cross-subject tasks.

## 1. Introduction

Machine learning (ML) has become a new standard in brain signals analysis (Hramov et al., 2021). ML is a model-free approach that successfully operates with data without prior knowledge of its origin. When the mathematical model of the time series is unknown, ML can build this model based on training data. Thus, being trained on a representative amount of data, ML enables the classification, detection, and prediction of the newly acquired data. These aspects of ML meet the fundamental requirements for brain-computer interfaces (BCIs). First, BCI often utilizes brain activity biomarkers that barely have an exact mathematical model. Second, brain activity varies between and within subjects; therefore, if a model exists, it changes unpredictably. Finally, ML demands low computational costs. Once trained, ML analyzes data very fast, even on mobile computers and smartphones.

In a classical paradigm, BCI operators participate in a calibration session to accumulate training data (Shenoy et al., 2006). They perform a series of predefined tasks to produce brain data, for which their intentions are known. ML uses this labeled data to learn associations between brain states and intentions. After the training, operators can control the BCI and improve their performance through feedback. Numerous studies used different feedback paradigms and reported their positive effect on the performance of BCI operators (Barsotti et al., 2017; Zapała et al., 2018; Abu-Rmileh et al., 2019; Duan et al., 2021). In the recent study (Duan et al., 2021), the authors introduced an online data visualization feedback protocol that intuitively reflects the EEG distribution in real-time. The results showed favorable training effects in terms of class distinctiveness and EEG feature discriminancy. Another study (Zapała et al., 2018) tested different approaches to visual feedback training and demonstrated positives effects of all of them. In Abu-Rmileh et al. (2019), the authors proposed using coadaptive feedback training in which the brain and the machine need to adapt in order to improve performance. Using this approach for the motor imagery BCI, the authors demonstrated improving the performance. Unlike the visual feedback, Barsotti et al. used vibration-evoked kinaesthetic feedback for motor imagery BCI and reported improvement of the BCI performance (Barsotti et al., 2017).

This review includes few recent studies reporting the positive effect of feedback on BCI operator performance. Their general idea is that the operator evaluates the correctness of their intentions in real-time. For instance, in the motor imagery (MI)-based BCI, a cursor moving to the left or right reflects the imagery movements of these hands. The feedback session can improve the decoder performance, but unable to train it from scratch. To utilize feedback, the decoder should demonstrate a reasonable accuracy of translating the intentions of the operator into BCI commands. The classical BCI protocols address this issue by training the decoder during the calibration session before starting the feedback and the further test sessions. This approach assumes that the input points in the calibration set follow the same probability distribution as the input points in the future feedback phase. At the same time, this assumption is usually not satisfied (Sugiyama et al., 2007). For some subjects, this problem is successfully addressed in a supervised fashion, i.e., by using the first trials from the feedback session. An alternative approach is using an adaptive learning strategy that combines supervised and unsupervised learning (Lu et al., 2009). As BCIs transit from laboratory settings into daily life, an important goal becomes minimizing the reliance of decoding algorithms on the calibration or enabling calibration with minimal burden on the user. One of the potential solutions is a pre-trained ML that demonstrates a reasonable accuracy on the newly-recruited operators.

We suppose that a pre-trained classifier should use EEG features corresponding to the fundamental neurophysiological processes, common for all subjects (Hramov et al., 2017, 2018, 2019; Maksimenko et al., 2018b). To reveal these features, we propose using statistical testing of the EEG spectral power between the classes in the representative group of subjects.

We tested our hypothesis using the visual stimuli classification task, where the convolutional neural network (CNN) classified two-dimensional EEG scalp topograms corresponding to processing visual stimuli with low (class 1) and high (class 2) ambiguity. First, we selected time-frequency features using within-subject statistical contrast between the classes. Thus, we suggested that revealed biomarkers referred to the fundamental neural processes shared between subjects. Utilizing these features, CNN trained on 19 subjects could classify data of a new participant with 74% accuracy. When we excluded a particular participant from the feature extraction procedure, the time-frequency features changed. For both time and frequency bands, change grew when the statistical significance of features was low. The classification accuracy remained stable against changes in the frequency band but decreased when the time-band changed.

These results suggest the effectiveness of our approach to ML training if the statistical contrast of selected features between the classes reaches a high significance. Simultaneously, the accuracy may decrease as the time-bands change due to inter-subject variability and the coexistence of different neural processes that rapidly replace each other. We expect that the effect of time-bands diminishes when considering slow processes during the resting state.

Finally, we put our results in the context of transfer learning (TL), a ML paradigm that addresses the cross-subject problem in BCI. In terms of TL, we referred representative group of subjects to as the source domain. The remaining test subject represented the target domain. We demonstrated that our approach enabled extracting interpretable feature subspace from the source data related to the target data, preventing the negative transfer in the cross-subject tasks.

## 2. Methods

### 2.1. Neurophysiological Data

We used experimental data collected in the Neuroscience and Cognitive Technology Lab at the Innopolis University (Innopolis, Russia) following the Declaration of Helsinki and the local Research Ethics Committee. Our recent studies (Maksimenko et al., 2020b, 2021) provide a detailed description of the experimental procedures, while the acquired EEG and behavioral data are available online from Figshare.com (Maksimenko et al., 2020a).

During the experiment, 20 healthy volunteers (16 men aged 20–36) sat in a comfortable chair with the two-button keypad in their hands. We repeatedly presented ambiguous stimuli, Necker cubes on the computer screen in front of them. The stimulus presentation time varied from 1 to 1.5 s. The pause between the presentations was 3–5 s. Presentation time and pauses were randomized through the experiment. We instructed participants to define the orientation of each stimulus and report their choice using the joystick. The left and right buttons stood for the left and the right orientations.

**Figure 1:**
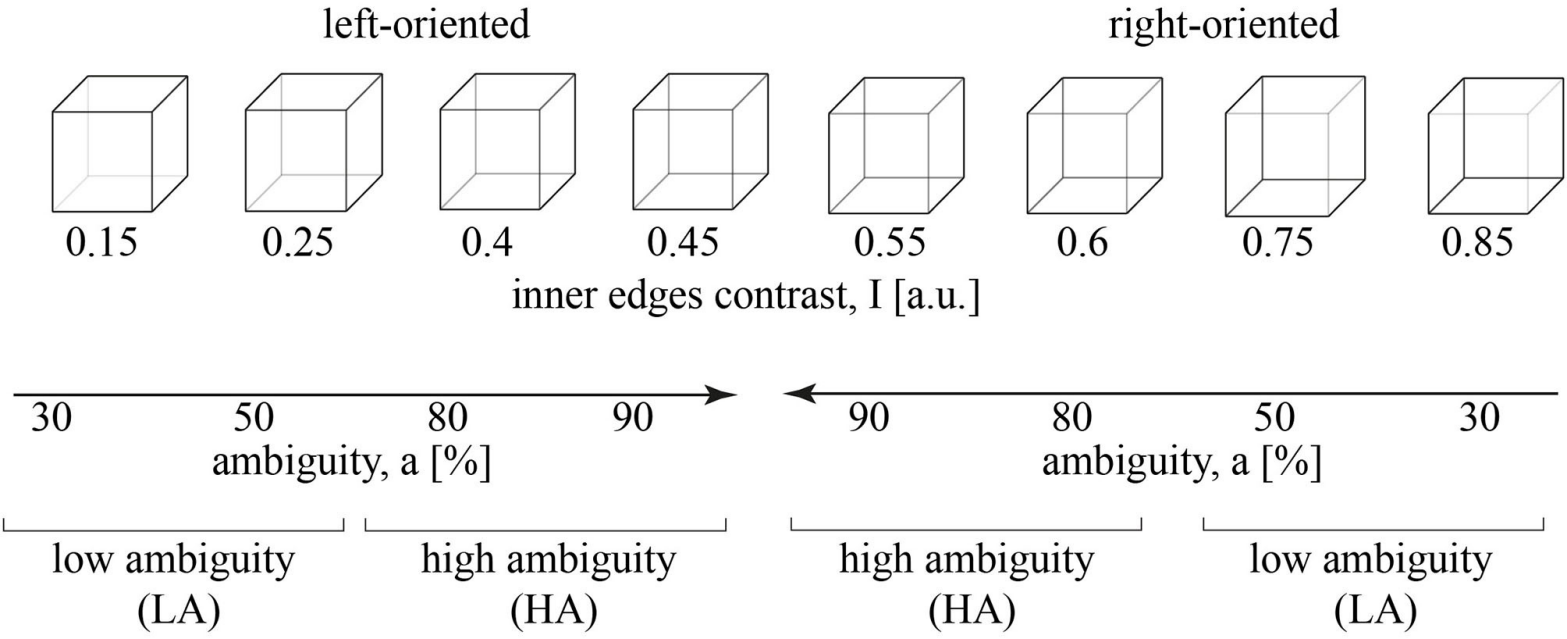
A set of visual stimuli, Necker cubes, with the contrast of the inner edges, *I*. The stimuli with *I* = 0.15, 0.25, 0.4, 0.45 are oriented to the left, while the stimuli with *I* = 0.55, 0.6, 0.75, 0.85 are oriented to the right. The inner edges contrast defines a stimulus ambiguity, *a* that varies from 30 to 90%. *a* = 30%, 50% corresponds to the low ambiguity (LA) and *a* = 80%, 90% - to the high ambiguity (HA).

Figure 1 illustrates the set of visual stimuli—Necker cube images with the different contrast of the inner edges (Necker, 1832; Kornmeier and Bach, 2005). For each cube, we introduced parameter *I* = 0.15, 0.25, 0.4, 0.45, 0.55, 0.6, 0.75, 0.85 defining the inner edges contrast. It reflected the intensity of three lower-left lines, while 1 − *I* corresponded to the intensity of three upper-right lines. The parameter *I* can be defined as *I* = 1 − *y*/255, where y is the brightness level of three lower-left lines using the 8-bit gray-scale palette. The value of *y* varies from 0 (black) to 255 (white) (Maksimenko et al., 2018a). Then we introduced stimulus ambiguity, a in the following way. We supposed that for *I* = 0, the stimulus is unambiguously left-oriented, while for *I* = 0.5, its features barely reflect the orientation. Varying *I* from 0 to 0.5, we increase the ambiguity of the left-oriented cube making it totally ambiguous at *I* = 0.5. Thus, setting *a* = 0 ambiguity for *I* = 0 and *a* = 100% ambiguity for *I* = 0.5, we suggest that stimuli with *I* = 0.15, 0.25, 0.4, 0.45 correspond to *a* = 30%, 50%, 80%, 90% ambiguity. Similarly, for the right-oriented stimuli, we obtain that cubes with *I* = 0.85, 0.75, 0.6, 0.55 also correspond to *a* = 30%, 50%, 80%, 90% ambiguity. Finally, to exclude effects of the stimulus orientation (including the effects associated with the formation of the motor response), we combined left- and right-oriented stimuli for each ambiguity.

Similar to our recent study (Kuc et al., 2021), we reduced the number of experimental conditions considering *a* = 30%, 50% as the low ambiguity (LA) stimuli and *a* = 80%, 90% as high ambiguity (HA) stimuli. Each group included 100 stimuli (25 per ambiguity, 50 per orientation). This simplification was based on our previous studies on the Necker cube images (Maksimenko et al., 2020b, 2021). It enabled revealing effects of ambiguity and provided a sufficient number of trials to minimize additional effects of orientation, a bias of the presentation moment, and the previously presented stimulus (Maksimenko et al., 2021).

### 2.2. Data Processing Pipeline

**Figure 2:**
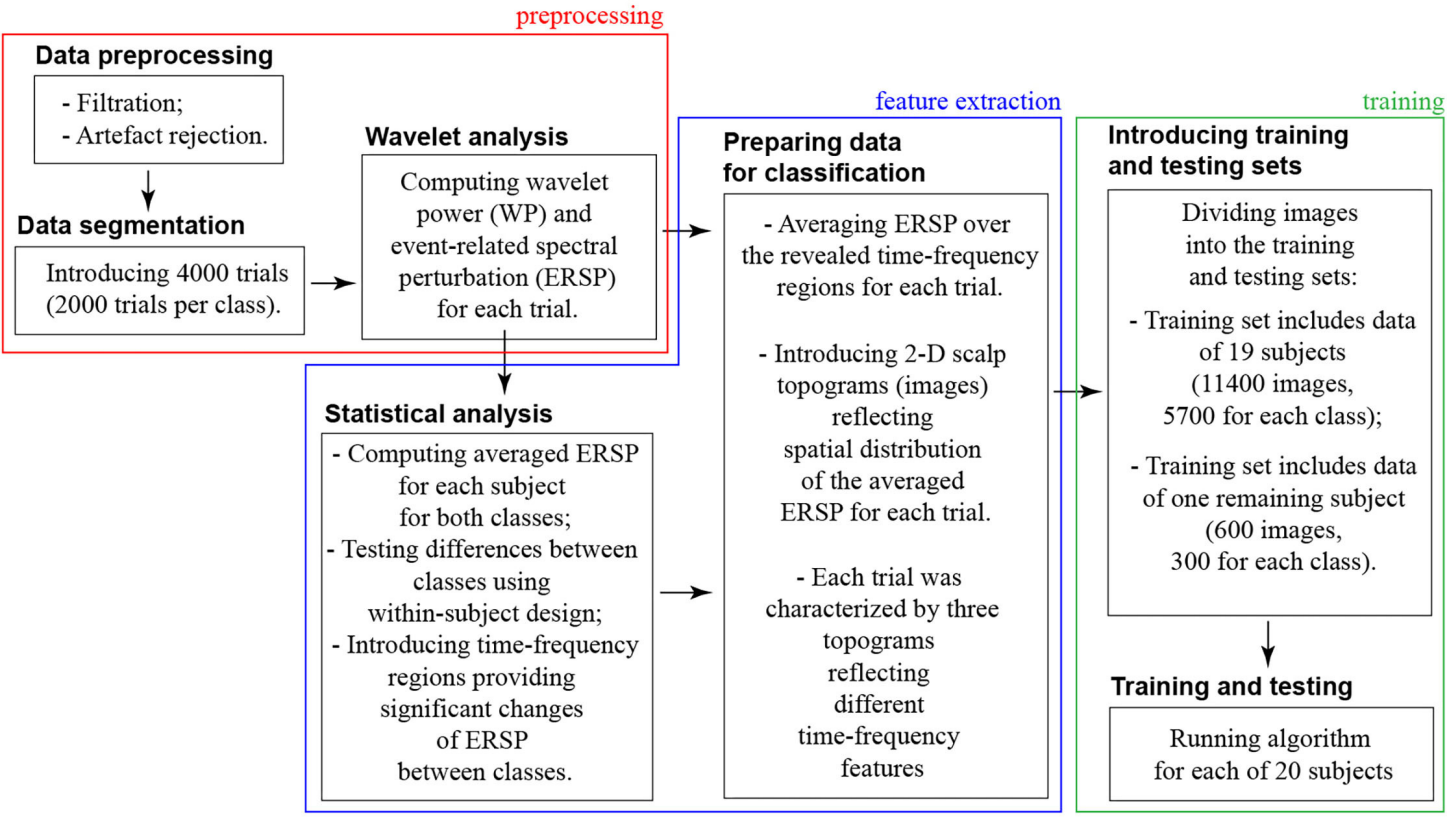
Scheme of the data processing pipeline.

We organized the data processing into three blocks: preprocessing → feature extraction → training (refer to Figure 2).

In the **preprocessing block**, we dealt with the raw continuously recorded EEG signals. Thus, the preprocessing procedure included artifacts rejection, segmentation data into the trials, and wavelet analysis.

- First, we filtered raw EEG signals by a band-pass FIR filter with cut-off points at 1 and 100 Hz and a 50-Hz notch filter. Second, we removed Eye-blinking artifacts using Independent Component Analysis (ICA) in the EEGLAB software (Delorme and Makeig, 2004). The EEG dataset of 31 channels was decomposed into 31 independent components using the “runica” function. To determine components with artifacts, we examined their scalp map projections, waveforms, and spectra. The components containing Eye-blinking artifacts usually had the leading positions in the component array due to high amplitude. They demonstrated a smoothly decreasing spectrum and their scalp map showed a strong far-frontal projection. Finally, Eye-blinking artifacts had the typical waveform; therefore, those segments of EEG signals were marked by the experienced neurophysiologist and used for determining the corresponding independent components. We removed the component with artifacts by using the Remove component tool.
- Then, we introduced 4-s EEG trials time-locked to the stimulus onset, including 2-s prestimulus and 2-s post-stimulus segments. Time-locking EEG signals to the stimulus onset, we focused on the processes that prevailed after the stimulus onset. We referred them to as the stimulus processing stage. In general, stimulus processing involves processing and decision-making stages (Siegel et al., 2011). The processing stage takes place in the occipital cortex during 130–320 ms post-stimulus onset, and the decision-making stage lasts longer and activates parietal and frontal areas (Mostert et al., 2015). Thus, while this study focuses on the processing stage, analysis of the decision-making process may require time-locking EEG signals to the moment of behavioral response.
- For each trial, we calculated wavelet power (WP) in the frequency band of 4–40 Hz using the Morlet wavelet. The Morlet wavelet *W*(*f, t*) is the product of a complex sine wave and a Gaussian function:
  (1) $$\begin{array}{l} W\left(f,t\right)=e^{2i\pi ft}\times e^{-t^{2}/2\sigma^{2}}, \end{array}$$
  where *i* is the imaginary operator, *f* is the frequency in Hz, *t* reflects the time in seconds, and σ is the Gaussian width, defined as
  (2) $$\begin{array}{l} \sigma =n/2\pi f. \end{array}$$
  The parameter *n* called the number of cycles defines the time-frequency precision trade-off. For neurophysiology data such as EEG, and MEG, *n* varies from 2 to 15 over frequencies between 2 and 80 Hz (Cohen, 2019). We defined *n* for each frequency *f* as *n* = *f*. The wavelet analysis was performed in Matlab using the Fieldtrip toolbox (Oostenveld et al., 2011). We considered WP on the 1-s interval, including 0.5-s prestimulus and 0.5-s post-stimulus segments.
- Finally, we calculated event-related spectral perturbation (ERSP) by contrasting post-stimulus WP to the prestimulus WP as
  (3) $$\begin{array}{l} ERSP=\frac{\text{poststimulus WP }-\text{ prestimulus WP }}{\text{prestimulus WP }}. \end{array}$$
  The obtained ERSP represent average spectral changes in response to a stimulus at each time moment during the 0.5-s post-stimulus epoch and at each frequency (Grandchamp and Delorme, 2011).

In the **feature extraction block**, we contrasted ERSP between two classes (HA and LA stimuli) to specify the time-frequency ranges providing a significant change of ERSP between classes. We organized this analysis in the following steps:

- For each subject, we averaged the ERSP over 100 trials corresponding to HA and LA stimuli. Thus, we obtained ERSP_LA_(*ch, f, t*) and ERSP_HA_(*ch, f, t*), where *ch* = 1…31 is the number of EEG channel, *f* ∈ [4, 40] Hz—frequency, and *t* ∈ [−0.5, 0.5] s reflects the time related to the stimulus onset.
- We compared ERSP_LA_(*ch, f, t*) and ERSP_HA_(*ch, f, t*) in the group of participants using a paired-samples *t*-test in conjunction with the cluster-based correction for multiple comparisons (Maris and Oostenveld, 2007). Specifically, we performed *t*-tests to compare each pair of the (channel, frequency, and time)-triplets. Elements that passed a threshold value corresponding to a *p*-value of 0.01 (two-tailed) were marked together with their neighboring elements and were collected into separate negative and positive clusters. The minimal number of required neighbors was set to 2. The *t*-values within each cluster were summed and rectified. These values were fed into the permutation framework as the test statistic. A cluster was considered significant when its *p*-value was below 0.025, corresponding to a false alarm rate of 0.01 in a two-tailed test. The number of permutations was 2,000. Analysis was performed in the Fieldtrip toolbox for Matlab.
- Statistical analysis provided us with the subspaces in the (channel, frequency, and time) domain where the differences between ERSP_LA_(*ch, f, t*) and ERSP_HA_(*ch, f, t*) were significant. We referred these subspaces to as clusters. For the *i*-th cluster, we specified the frequency band [$f_{i}^{1};f_{i}^{2}$] and the time interval [$t_{i}^{1};t_{i}^{2}$]. For each stimulus, we averaged ERSP over this time-frequency range and plotted its distribution on the scalp topogram using the Fieldtrip toolbox. The final set of 2-D topograms is available from the public repository (Maksimenko and Kuc, 2021).

In the **training and testing block**, we used an ML algorithm to classify 2-D topograms corresponding to HA and LA stimuli processing.

**Figure 3:**
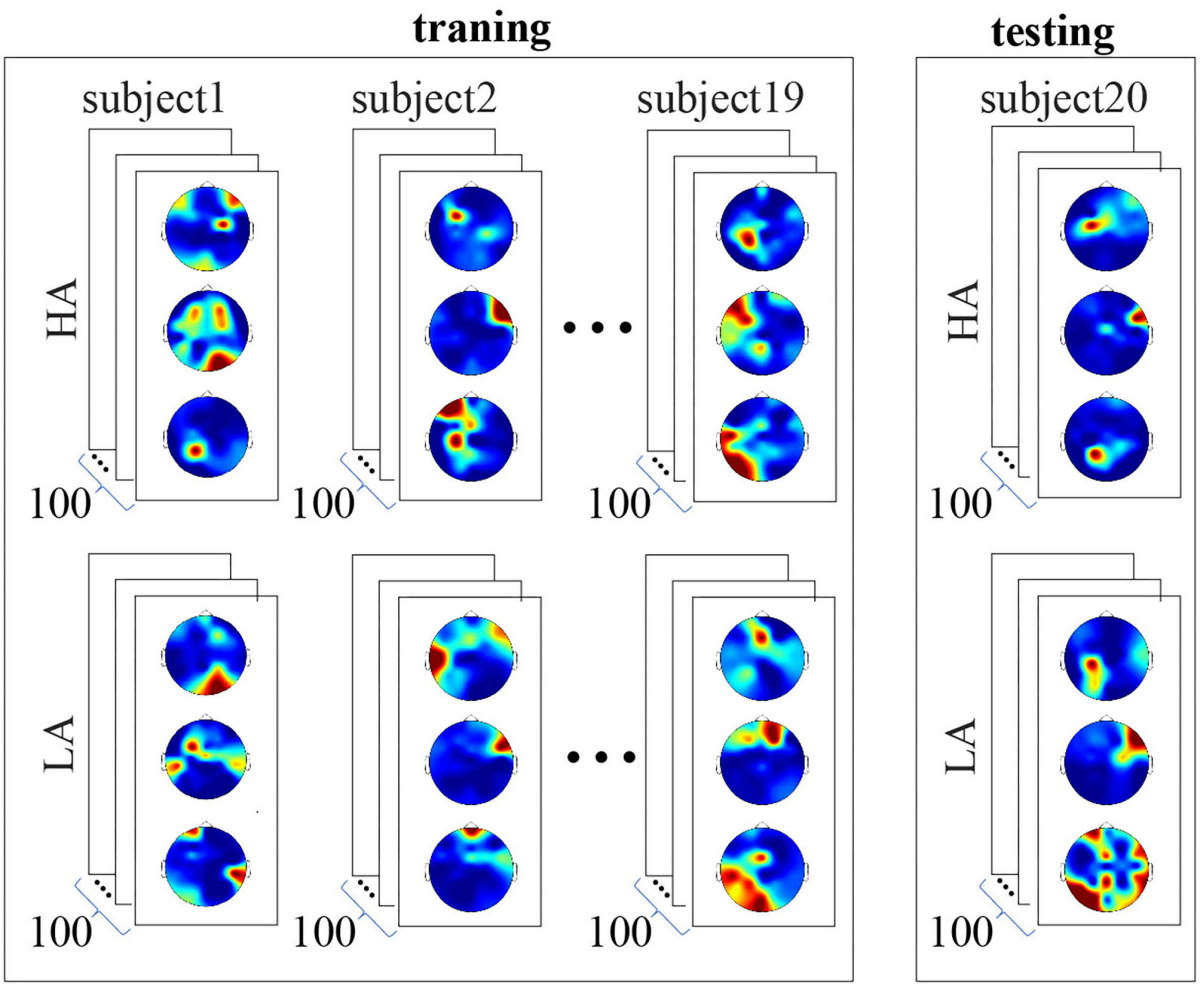
Structure of the training and testing datasets. The training set includes data of 19 subjects, 11,400 images in total. Each subject's data includes 600 images, 300 LA, and 300 HA topograms. The testing set includes data of the remaining single subject. It consists of 600 images, 300 LA, and 300 HA topograms. Convolutional neural network (CNN) did not learn the data of the test subject.

- We used a CNN with a Resnet 50 topology (He et al., 2016). ReLu was an activation function. We implemented CNN in Python using the TensorFlow library, and Keras module. Image size was reduced to 224 × 224 pixels using the Image Rescaling procedure (Xiao et al., 2020). We used the backpropagation method to train CNN.
- As shown above, three scalp topograms characterized neural activity during the stimulus processing. Each subject perceived 100 LA and 100 HA stimuli; therefore, each subject's data included 600 images (300 LA and 300 HA topograms). We included 11,400 of 19 subjects in the training set. The testing set consisted of 600 images belonging to one subject (refer to Figure 3). Thus, CNN did not learn the data of the test subject. This procedure was repeated 20 times to test CNN for each subject. Cross-entropy served a loss function.
- Finally, we utilized Adam's optimizer to select the optimal parameters of the neural network (Zhang, 2018). To evaluate the CNN performance, we analyzed the traditional metrics, accuracy, precision, and recall. The source code is available online at Google Colab, https://colab.research.google.com/drive/1VpyiU66Xy6wNLgd_7OR1QbX4fE4iAFw6 from S. Korchagin under request.

### 2.3. Data Analysis

To test how the time and frequency ranges changed when excluding one subject from the feature extraction procedure, we used a repeated-measures ANOVA. We ran tests separately for the frequency and time and different clusters. For frequencies, we defined within-subject factors as the frequency-bounds ($f_{i}^{1}$ and $f_{i}^{2}$) and the type of feature extraction procedure (all subjects included vs. one subject excluded). For times, within-subject factors included the time-bounds ($t_{i}^{1}$ and $t_{i}^{2}$) and the type of feature extraction procedure. First, we tested the main effects. For the significant main effects, we performed a *post-hoc* *t*-test to evaluate the effect direction.

For each of subject, we quantified the changes of the frequency Δ*f*_*i*_ and time Δ*t*_*i*_ intervals as


(4) $$\begin{array}{l} \Delta f_{i}=\frac{\left|{\left(f_{i}^{1}\right)}_{ind}-{\left(f_{i}^{1}\right)}_{com}|+|{\left(f_{i}^{2}\right)}_{ind}-{\left(f_{i}^{2}\right)}_{com}\right|}{2\left[{\left(f_{i}^{2}\right)}_{com}-{\left(f_{i}^{1}\right)}_{com}\right]}\times 100\%, \end{array}$$

(5) $$\begin{array}{l} \Delta t_{i}=\frac{\left|{\left(t_{i}^{1}\right)}_{ind}-{\left(t_{i}^{1}\right)}_{com}|+|{\left(t_{i}^{2}\right)}_{ind}-{\left(t_{i}^{2}\right)}_{com}\right|}{2\left[{\left(t_{i}^{2}\right)}_{com}-{\left(t_{i}^{1}\right)}_{com}\right]}\times 100\%, \end{array}$$

where *i* is the number of clusters, |...| reflects the absolute value. The subscript *com* defines the time-frequency values obtained when all subjects participated in the statistical analysis. The subscript *ind* corresponds to the case when one subject was excluded.

To test how Δ*f*_*i*_ and Δ*t*_*i*_ changed between clusters, we used a repeated-measures ANOVA. The cluster number (*i* = 1, 2, 3) and the type of change (Δ*f*_*i*_ and Δ*t*_*i*_) served as within-subject factors. First, we tested the main effects of all factors and their interactions. For the significant effects, we performed a *post-hoc* *t*-test to evaluate the effect direction.

To test whether CNN accuracy depended on Δ*f*_*i*_ and Δ*t*_*i*_, we conducted the multiple regression analysis. We built a separate regression model for Δ*f*_*i*_ and Δ*t*_*i*_. The CNN accuracy was an independent variable, and Δ*f*_*i*_ and Δ*t*_*i*_ served as predictors.

## 3. Results

**Figure 4:**
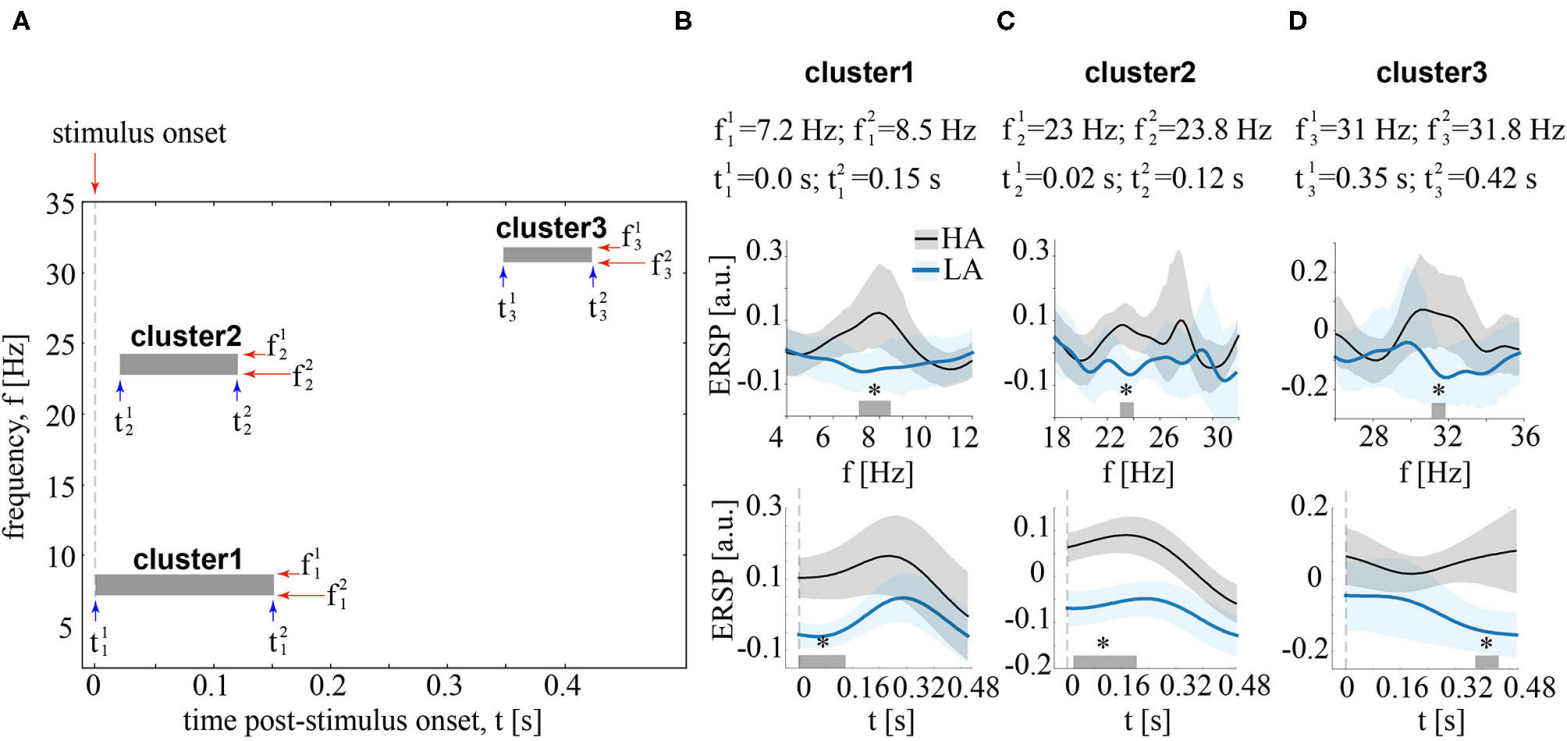
**(A)** Boxes show clusters in the time-frequency domain reflecting the significant differences of EEG power, event-related spectral perturbation (ERSP) between HA and LA stimuli. **(B–D)** contain information about the time and frequency ranges of each cluster and show ERSP averaged over these time (upper row) and frequency (bottom row) ranges. ERSP is shown for LA and HA stimuli as the group means and 95% confidence intervals, ^*^p < 0.05 *via* the paired *t*-test, corrected *via* a cluster-based approach with 2000 Monte-Carlo randomizations.

Contrasting ERSP between HA and LA stimuli in the time-frequency domain, we observed three significant positive clusters with *p* < 0.01 as shown in Figure 4A. The first cluster extended from $t_{1}^{1}=0$ s to $t_{1}^{2}=0.150$ s post-stimulus onset for the frequencies ranged from $f_{1}^{1}=7.25$ Hz to$f_{1}^{2}=8.5$ Hz (refer to Figure 4B). The ERSP in this cluster was higher for HA stimuli in 18 subjects. The second cluster extended from $t_{2}^{1}=0.02$ s to $t_{2}^{2}=0.2$ s post-stimulus onset for the frequencies from $f_{2}^{1}=23$ Hz to $f_{2}^{2}=23.8$ Hz (refer to Figure 4C). According to the distribution of pairwise differences, this cluster had higher ERSP for HA stimuli in 17 subjects. The third cluster extended from $t_{3}^{1}=0.35$ s to $t_{3}^{2}=0.42$ s. Its frequency range extended from $f_{3}^{1}=31$ Hz to $f_{3}^{2}=31.8$ Hz (Figure 4D). Sixteen subjects demonstrated higher ERSP for HA stimuli in this cluster.

We collected the frequency bands [$f_{i}^{1};f_{i}^{2}$] and the time intervals [$t_{i}^{1};t_{i}^{2}$] of these clusters. They served as a set of features containing the most pronounced differences between HA and LA stimuli in the group of subjects. We used these features to train CNN. As a result, CNN accuracy varied from 71% to 76% (*M* = 74%, *SD* = 1.6%). Precision varied from 70 to 75% (*M* = 73%, *SD* = 1.9%). Recall varied from 63% to 71% (*M* = 67%, *SD* = 2.5%).

Second, we tested how the time-frequency ranges of each cluster changed when excluding one subject from the feature extraction procedure.

**Figure 5:**
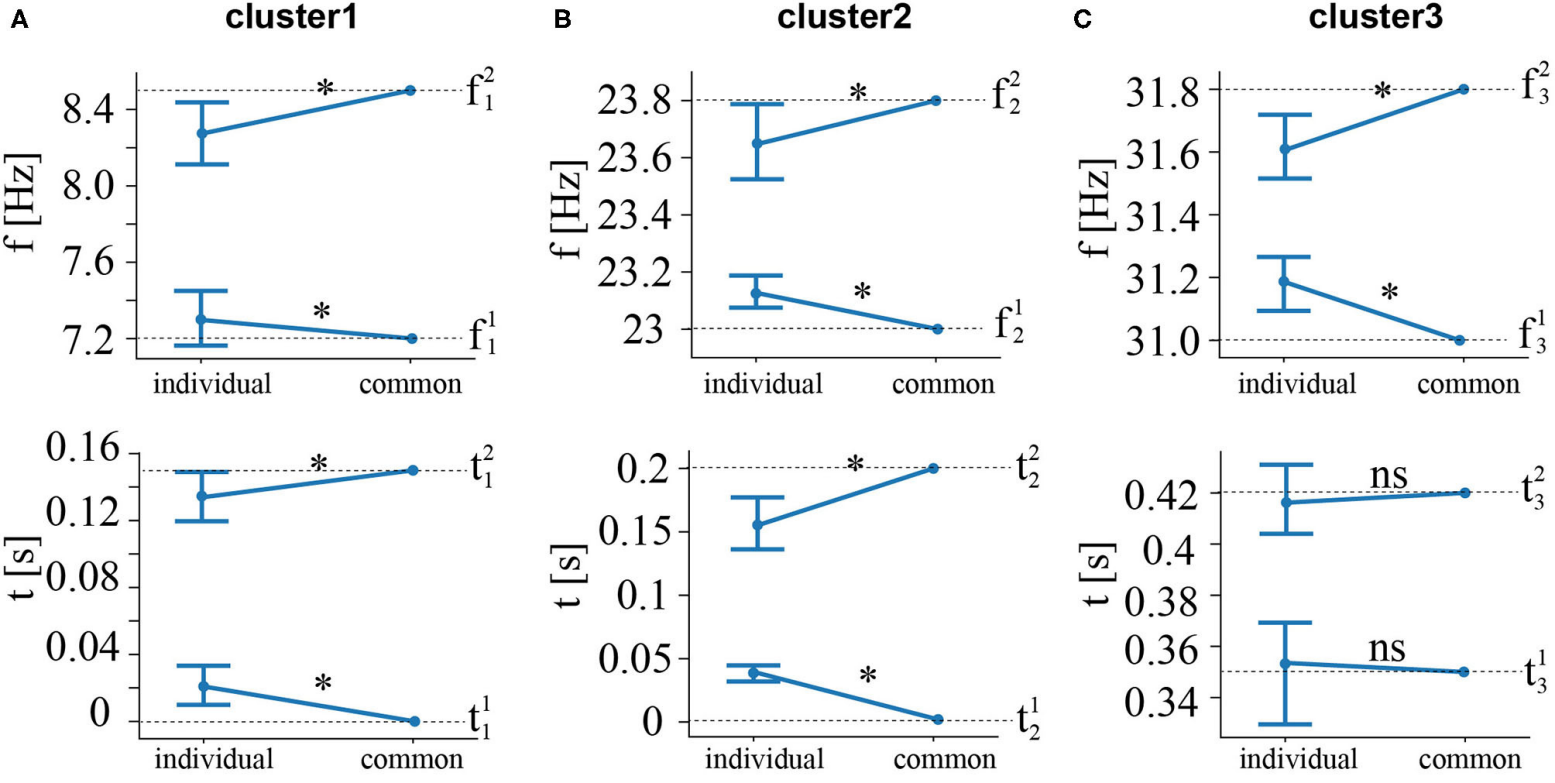
Changes in the frequency (upper row) and time (bottom row) ranges when one subject is excluded from the feature selection procedure. The **(A–C)** correspond to different clusters. Frequencies ${\left(f_{1,2}^{i}\right)}_{common}$ and times ${\left(t_{1,2}^{i}\right)}_{common}$ are obtained when including all 20 subjects. The values ${\left(f_{1,2}^{i}\right)}_{individual}$ and ${\left(t_{1,2}^{i}\right)}_{individual}$ reflect the time-frequency bands obtained when one subject is excluded. ${\left(t_{1,2}^{i}\right)}_{individual}$ are shown as group mean and 95% confidence interval. ^*^p < 0.05, *via* a repeated measures ANOVA and a *post-hoc* *t*-test.

For cluster 1 (Figure 5A), the main effect of the feature extraction procedure on frequencies was insignificant: *F*_(1, 19)_ = 2.994, *p* = 0.1. At the same time, we observed a significant interaction effect frequency bound * feature extraction procedure: *F*_(1, 19)_ = 4.8, *p* = 0.041. The *post-hoc* analysis revealed that *f*^1^ remained unchanged: *t*_(19)_ = 0.639, *p* = 0.53. In contrast, *f*^2^ decreased (*M* = 8.27 Hz, *SD* = 0.37) when one subject was excluded from the analysis: *t*_(19)_ = −2.7, *p* = 0.14. The main effect of the feature extraction procedure on time was also insignificant: *F*_(1, 19)_ = 0.37, *p* = 0.55. There was a significant interaction effect time bound * feature extraction procedure: *F*_(1, 19)_ = 10.9, *p* = 0.004. The *post-hoc* analysis revealed that $t_{1}^{1}$ increased (*M* = 0.021 s, *SD* = 0.027) when one subject was excluded from the analysis: *t*_(19)_ = 3.41, *p* < 0.003. In contrast, $t_{2}^{2}$ did not change (*M* = 0.13 s, *SD* = 0.03): *t*_(19)_ = −1.93, *p* = 0.069.

For cluster 2 (Figure 5B), the main effect of the feature extraction procedure on frequencies was insignificant: *F*_(1, 19)_ = 0.101, *p* = 0.755. At the same time, we observed a significant interaction effect frequency bound * feature extraction procedure: *F*_(1, 19)_ = 13.6, *p* = 0.002. The *post-hoc* analysis revealed that $f_{2}^{1}$ increased (*M* = 23.12 Hz, *SD* = 0.12) when one subject was excluded from the analysis: *t*_(19)_ = 0.639, *p* = 0.53. In contrast, $f_{2}^{2}$ decreased (*M* = 23.65 Hz, *SD* = 0.32), but has the weak statistical power: *t*_(19)_ = −2.1, *p* = 0.49. The main effect of the feature extraction procedure on time was significant: *F*_(1, 19)_ = 5.59, *p* = 0.029. Simultaneously, there was a significant interaction effect time bound * feature extraction procedure: *F*_(1, 19)_ = 28.59, *p* < 0.001. The *post-hoc* analysis revealed that $t_{2}^{1}$ increased (*M* = 0.039 s, *SD* = 0.014) when one subject was excluded from the analysis: *t*_(19)_ = 5.66, *p* < 0.001. In contrast, $t_{2}^{2}$ decreased (*M* = 0.15 s, *SD* = 0.04): *t*_(19)_ = −4.08, *p* = 0.001.

Cluster 3 (Figure 5C) was not observed in four subjects. For the rest of the subjects, we ran ANOVA in a similar way with clusters 1 and 2. Again, we found an insignificant main effect of the feature extraction procedure on frequencies: *F*_(1, 15)_ = 0.004, *p* = 0.953, and a significant interaction effect frequency bound * feature extraction procedure: *F*_(1, 15)_ = 20.01, *p* < 0.001. The *post-hoc* analysis revealed that $f_{3}^{1}$ increases (*M* = 31.18 Hz, *SD* = 0.17): *t*_(15)_ = 4.392, *p* = 0.001. In contrast, $f_{3}^{2}$ decreased (*M* = 31.6 Hz, *SD* = 0.22) when one subject was excluded from the analysis: *t*_(19)_ = −3.419, *p* = 0.004. Finally, The main effect of the feature extraction procedure on time was insignificant: *F*_(1, 15)_ = 0.001, *p* = 0.971, as well as an interaction effect time bound * feature extraction procedure: *F*_(1, 15)_ = 0.264, *p* = 0.615.

**Figure 6:**
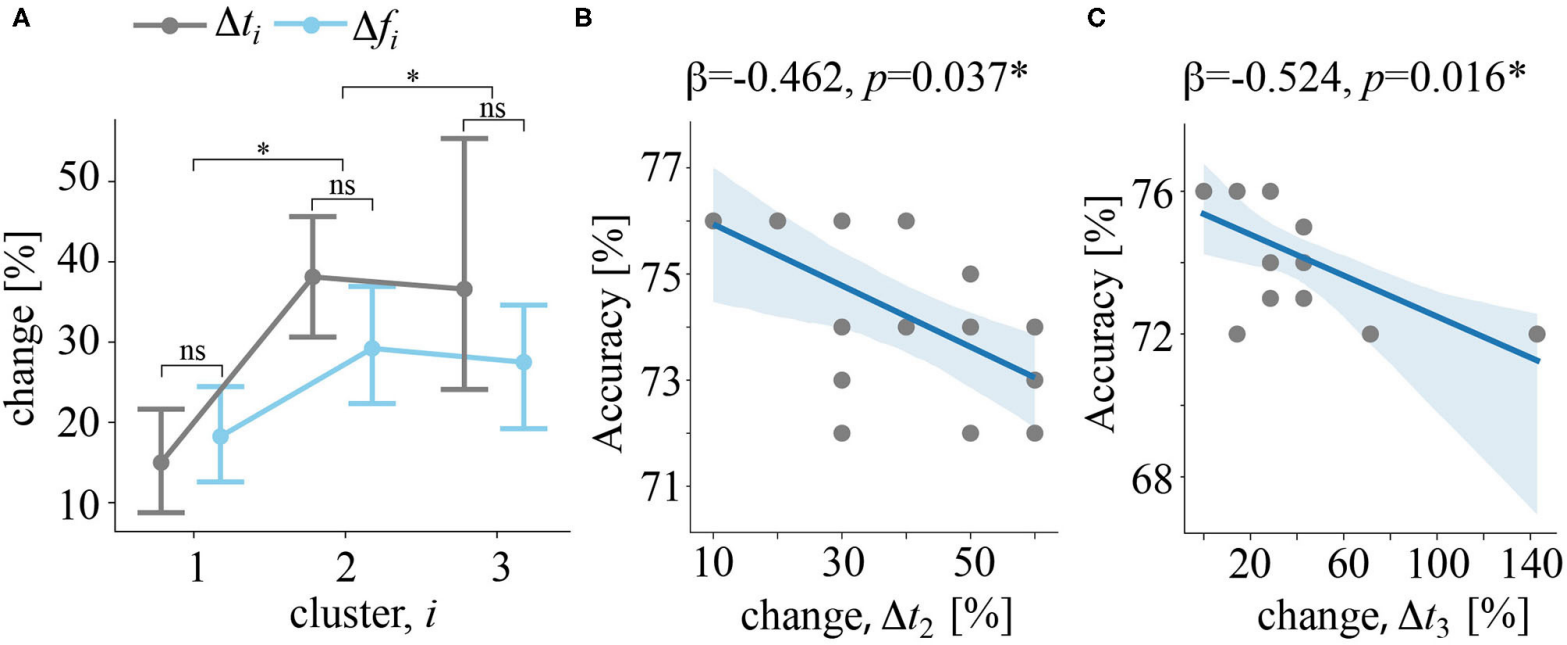
**(A)** Δ*f*_*i*_ and Δ*t*_*i*_ quantify how the frequency and the time band changed when excluding a particular subject from the feature selection procedure, *i* is the cluster number. Data are shown as the group mean and 95% confidence interval, ^*^p < 0.05, and ^ns^*p* > 0.05 *via* ANOVA and a *post-hoc t*-test. **(B)** The regression plot reflects the negative correlation between changes in the time band for cluster 2 and CNN classification accuracy. **(C)** Regression plot reflects the negative correlation between changes in the time band for cluster 3 and CNN classification accuracy. Dots show the data of single subjects. The solid line is a regression model. The shadow area illustrates the 95% confidence interval.

For each of 16 subjects having all three clusters, we quantified the change of the frequency Δ*f*_*i*_ and time Δ*t*_*i*_ intervals (refer to Equation 5 in Methods). First, we tested how these changes depends on the cluster number. As a result, we reported a significant main effect of cluster number: *F*_(2, 30)_ = 5.697, *p* = 0.008, insignificant effect of the change type: *F*_(1, 15)_ = 2.317, *p* = 0.149, and insignificant effect of their interaction: *F*_(2, 30)_ = 1.621, *p* = 0.215. Together, these results show that change in both frequency and time increased with the cluster number (Figure 6A). At the same time, the change of time and frequency parameters were similar. Second, we tested whether these changes predict CNN accuracy. We found, that Δ*t*_*i*_ statistically significantly predicted CNN accuracy: $F_{\left(3,12\right)}=5.87,p=0.01,R^{2}=0.595$. We found that Δ*t*_2_ (β = −0.462, *p* = 0.037) (Figure 6B) and Δ*t*_3_ (β = −0.524, *p* = 0.016) (Figure 6C) significantly predicted CNN accuracy. The value of Δ*t*_1_ was unable to predict CNN accuracy (*p* = 0.711). Finally, Δ*f*_*i*_ failed to predict accuracy rate: $F_{\left(3,12\right)}=0.745,p=0.546,R^{2}=0.157$.

## Discussion

In this study, we tested whether CNN trained on some subjects' data could classify data of a new subject. We used an EEG dataset obtained during perception of visual stimuli with the LA and HA degrees and defined LA and HA as two classes. According to the literature, these two classes exhibit distinctive features of neural activity. Thus, LA stimuli processing depends on the stimulus morphology and relied on the bottom-up mechanism. In contrast, the morphology of HA stimuli has much less information; therefore, subjects relied on the top-down processes to unresolved ambiguity of the visual signal (Kuc et al., 2021; Maksimenko et al., 2021).

We hypothesized that features of LA and HA stimuli processing referred to the basic neurophysiological mechanisms, common for a large population of conditionally healthy subjects (Maksimenko et al., 2020b). To extract these features, we contrasted electroencephalographic (EEG) spectral power between LA and HA stimuli in a group of 20 volunteers. As a result, we observed three clusters representing significant changes between the two classes.

- In cluster 1, HA stimuli induced higher anterior theta-band power for 0.15 s post-stimulus onset. In line with previous studies, we treated it as a biomarker of top-down control (de Borst et al., 2012; Lee and D'Esposito, 2012; Cohen and Van Gaal, 2013), e.g., the prevalence of expectations and prior experience in ensuring correct perception when the sensory information is inconclusive (Mathes et al., 2014).
- In cluster 2, HA stimuli induced higher beta-band power over the occipito-parietal electrodes for 0.02–0.2 s post-stimulus onset. Previously, Yokota et al. (2014) linked beta-band activity with the interaction between occipital and parietal cortical regions, necessary for stimulus disambiguation.
- In cluster 3, HA stimuli induced higher beta-band band power over the parietal and midline frontal electrodes for 0.35–0.42 s post-stimulus onset. On the one hand, it might reflect the conscious processing of the perceptual information or maintenance of the percept in working memory (Pitts and Britz, 2011). On the other hand, fronto-parietal beta-band power might reflect decision-making (Chand and Dhamala, 2017; Spitzer and Haegens, 2017).

Thus, we revealed three time-frequency intervals carrying the biomarkers of top-down processes needed for HA stimuli processing. According to the literature, the different types of neural activity have distinctive topographical properties of the theta- and beta-band power. Thus, we reduced the feature space in the time-frequency domain and added features, describing topographical properties. As a result, each perception was characterized by three 2D images, illustrating the distribution of EEG power across the scalp and corresponding to three revealed significant clusters of neuronal activity.

Convolutional neural network learned to classify HA and LA stimuli using 2D images of 19 subjects. Then, it analyzed the data of the remaining subject. Performing this procedure for all volunteers, we obtained an accuracy rate of 74% ± 1.6 SD. Such small between-subject variability confirmed the shared nature of revealed biomarkers in the group.

Further, we tested the stability of the revealed time-frequency features against the exclusion of subjects on the stage of statistical testing. As a result, the first and second clusters appeared regardless of the subject exclusion. In contrast, the third cluster disappeared when excluding four subjects. All clusters changed in the way that the new time-frequency bands belonged to the former ones. We found no systematic shifts, backward or toward, on the frequency and time axis. These results also confirmed that the group-level clusters reflect shared features of brain activity. The first and the second clusters have higher statistical significance than the third cluster. It explained the absence of the third cluster when excluding four subjects.

Finally, we estimated how time and frequency bands changed when excluding a particular subject from the statistical analysis. We interpreted that degree of change quantified how well this subject followed group tendency. For both time and frequency bands, these changes grew with the cluster number. It confirmed that the high statistical significance of the cluster ensured the stability of its features against between-subject variability.

Then, we tested whether the degree of change explains the classification accuracy. We shown that neither frequency nor time changes in the first cluster did not predict accuracy. For the second and third clusters, changes in the frequency bands also did not affect the accuracy. In contrast, changes in the time intervals of the second and third clusters negatively correlated with the accuracy of the CNN-based classifier. We supposed that accuracy remained stable against changes in the frequency band but decreased when the time band changed. It might be evidence that spectral power changed in the frequency domain slower than in time. When the subject interpreted visual stimulus, different processes took place in the brain network, and one replaced the others inducing the dramatic changes in the spectral power.

The research issue under study is usually referred to as the cross-subject problem (Liu et al., 2020) and addressed in the framework of the TL approach (Pan and Yang, 2009).

In ML, TL is a paradigm that implies storing knowledge gained while solving one problem (referred to as a source domain) and applying it to a different but related problem (referred to as a target domain). For example, knowledge gained while learning to recognize cars could help to identify trucks. This feature of the TL enables addressing some critical issues in the BCI field. As discussed above, BCIs, especially those based on noninvasive signals, suffer from noise, artifact, and between-subject/within-subject non-stationarity. It severely hampers building a generic pattern recognition model, optimal for different subjects, during different sessions, for different devices and tasks. Using TL means that the decoder, which utilizes data or knowledge from similar or relevant subjects/sessions/devices/tasks to facilitate learning for a new subject/session/device/task.

A review study by Wu et al. (2020) describes the recent advances in using TL for addressing the main issues in EEG-based BCIs. According to the variations between the source and the target domains, they formulated four different TL scenarios for the EEG-based BCIs:

- Cross-subject TL uses data from the group of subjects (the source domain) to facilitate calibration for a new volunteer (the target domain). Usually, the task and EEG device are the same across subjects.
- Cross-session TL uses data from the prior sessions (the source domain) to facilitate calibration for a new session (the target domain). For example, data from previous days may facilitate the current calibration. Usually, the subject, task, and EEG device remain the same across sessions.
- Cross-device TL uses data from one EEG device (the source domain) to facilitate calibration for a new device (the target domain). Usually, the task and subject are the same across EEG devices.
- Cross-task TL uses data from similar or relevant tasks (the source domains) to facilitate calibration for a new one (the target domain). For example, data from left- and right-hand MI may facilitate calibration of the feet and tongue MI. Usually, the subject and EEG device are the same across tasks.

These TL scenarios utilize different TL methods. According to the recent review by Wan et al. (2021), they belong to four groups:

- Domain adaptation aims to improve the models to adapt to the data distribution in the target domain. It includes marginal distribution adaptation and conditional distribution adaptation. They benefit when the marginal or conditional distributions of the data in the source and target domains are different. Both methods facilitated EEG analysis in the cross-subject tasks.
- Subspace learning operates with EEG data as a dimensionality reduction technique. It aims to transform the source domain data and the target domain data into a subspace where their distributions are similar.
- Deep neural network (DNN) TL fine-tunes a pre-trained DNN model when no significant difference exists between the source and target domain. Fine-tuning may touch either the whole network or its layers. DNN TL is less effective when the distributions of the source domain and target domain are different. In this case, researchers resorted to DNN adaptation (DNA). It adjusts the cost function of the original network by adding a domain loss to measure the distribution of the source and target data.
- Improved common spatial patterns algorithms may reduce the difference of the data distribution between the source and target domains. They follow the hypothesis that both domains have features that are distributed similarly between them. A bulk of literature reported that using either standard or new CSP algorithms improves TL in EEG studies.

Despite the numerous successful applications of these TL approaches to EEG data, they all suffer from drawbacks. From its definition, TL implies transferring the knowledge between domains based on the relationship between the data. However, different EEG datasets demonstrate dissimilarity or the dependence between them is complicated. It causes the problem of negative transfer in EEG signal analysis. The negative transfer appears due to source-target data dissimilarity when the transfer method fails to find the transferable components (Novick, 1988). To avoid negative transfer, Wan et al. recommended analyzing the transferability between the source and target tasks before building ML models to guarantee the proper selection of the data sources and algorithms (Lin and Jung, 2017). In the DNN TL approaches, the literature review highlights other limitations. First, DNN methods lack interpretability and universality. Second, the network structure and parameters may affect the learning ability of DNN models.

One possible solution for preventing the negative transfer is extracting subspaces. These subspaces may carry the similarities between domains even if no relations exist between the initial data. Regarding EEG-based BCIs, we suppose that subspaces should include the fundamental EEG features shared between different subjects. For instance, in MI-based BCI, the user produces control commands utilizing mu-band event related desynchronization registered in the motor cortex (Grigorev et al., 2021). Another BCI paradigm uses the steady-state visual evoked potential (SSVEP) İşcan and Nikulin (2018). During a periodic stimulation with a frequency above 6 Hz, SSVEP is generated strongly in the occipital areas of the brain at the corresponding frequency. Thus, if the target stimulus exhibits periodic modulation, the occipital SSVEP will be registered once the user focuses on this stimulus. In a P300-based BCI, a series of repeating stimuli (e.g., letters) appear on a screen. For item selection, the user needs to attend to the target stimulus and ignore the rest. At the end of each sequence, the BCI identifies which stimulus was attended as P300 is expected to be generated for the target stimulus if well attended (Arvaneh et al., 2019). All these protocols of BCIs utilized the fundamental knowledge about brain functioning shared between different subjects.

If for the traditional BCI protocols the fundamental principles are known, the further development of BCIs may require detecting various brain states. For instance, designing the passive BCIs detecting a particular human state (fatigue, decreased attention, emotions, etc.) should utilize biomarkers differentiating these states from others. We suggest that these biomarkers represent the feature subspace carrying the statistically significant differences between the states.

Using within-subject statistical analysis, we found this subspace of features in the form of three time-frequency clusters with a clear neurophysiological interpretation. We assumed that these subspaces were shared between the subjects, even between those whose data were excluded from training. In terms of the TL, we extracted the subspace in a source domain (the representative group of subjects) and supposed its relation with the similar subspace of the target domain (new subject). Finally, we confirmed that the feature subspace extracted from the source domain contained one of the target domains. Having summarized, we supposed that our approach contributes to developing TL methods for BCI tasks. It enables extracting interpretable feature subspace from the source data related to the target data, preventing the negative transfer in the cross-subject tasks.

Finally, our study has potential limitations. The number of participants is small; therefore, there is a risk that a single subject will have features different from those defined for the group. We expect that including more participants in the feature selection procedure will diminish this risk. Second, we considered an unusual BCI protocol that differed from the traditional paradigms. Unlike most traditional protocols, e.g., MI, SSVEP, ERP, the EEG features (frequency and time) were unknown for this task. Thus, we used statistical analysis to extract the time-frequency subspaces from EEG signals reflecting the difference between classes. We suggest using this approach in BCIs that monitor human states involving complex cognitive processes. Further studies should consider the traditional BCI protocols to prove the universality of our approach.

## 4. Conclusion

Having summarized, we confirmed that CNN trained on 19 subjects could classify data of a new participant with 74% accuracy. We selected time-frequency EEG features using within-subject statistical contrast between the classes. Thus, we suggested that CNN utilized EEG biomarkers that referred to the fundamental neural processes shared between subjects. When we excluded a particular subject from the feature extraction procedure, the time-frequency features changed. For both time and frequency bands, change grew when the statistical significance of features was low. Finally, CNN accuracy remained stable against changes in the frequency band but decreased when the time-band changed.

These results suggest the potential of using our approach to ML training if the statistical contrast of selected features between the classes gives a high significance. Simultaneously, one must be careful about the changes of time-bands occurring due to inter-subject variability and the coexistence of different neural processes that rapidly replace each other. We expect that the effect of time-bands diminishes when considering slow processes during the resting state. To control the changes of time-bands, we advise adjusting time bands using optimization techniques.

Our results contribute to the BCI and ML fields. In the BCI field, a pre-trained classifier could help inexperienced users to start using BCI bypassing training and further increased accuracy during the feedback session. It may facilitate using BCI in paralysis or limb amputation when there is no explicit user-generated kinematic output to properly train a decoder. In the ML field, our approach may facilitate the development of TL methods for addressing the cross-subject problem. It allows extracting the interpretable feature subspace from the source data (the representative group of subjects) related to the target data (a naive user), preventing the negative transfer in the cross-subject tasks.

## Data Availability Statement

The datasets presented in this study can be found in online repositories. The names of the repository/repositories and accession number(s) can be found below: https://figshare.com/articles/dataset/Dataset_for_analysis_of_the_visual_stimulus_ambiguity_effect_on_the_behavioral_response_and_EEG_activity/12292637/2 and https://figshare.com/articles/dataset/Images_of_The_2D_EEG_Scalp_Topograms_Related_to_Ambiguous_Stimuli_Processing_for_training_the_convolutional_neural_network_/16645540.

## Ethics Statement

The studies involving human participants were reviewed and approved by Research Ethics Committee of Innopolis University. The patients/participants provided their written informed consent to participate in this study.

## Author Contributions

AH conceived and supervised the study. VM formulated the research hypothesis and methodology. AK and NS analyzed data. SK built a machine learning (ML) algorithm. VM and AH wrote the manuscript. All authors contributed to the article and approved the submitted version.

## Funding

VM has been supported by the Russian Foundation for Basic Research (Grant No. 19-32-60042) in formulating the research hypothesis and methodology. AK received support from President Grant (MK-1760.2020) in data analysis. AH received support from President Grant (NSH-2594.2020.2) in formulating research objectives. NS has been supported by the Ministry of Education and Science of the Russian Federation (State Assignment No. FZWM-2020-0013) in the literature review.

## Conflict of Interest

The authors declare that the research was conducted in the absence of any commercial or financial relationships that could be construed as a potential conflict of interest.

## Publisher's Note

All claims expressed in this article are solely those of the authors and do not necessarily represent those of their affiliated organizations, or those of the publisher, the editors and the reviewers. Any product that may be evaluated in this article, or claim that may be made by its manufacturer, is not guaranteed or endorsed by the publisher.
